# Tau deposition drives neuropathological, inflammatory and behavioral abnormalities independently of neuronal loss in a novel mouse model

**DOI:** 10.1093/hmg/ddv336

**Published:** 2015-08-13

**Authors:** Casey Cook, Silvia S. Kang, Yari Carlomagno, Wen-Lang Lin, Mei Yue, Aishe Kurti, Mitsuru Shinohara, Karen Jansen-West, Emilie Perkerson, Monica Castanedes-Casey, Linda Rousseau, Virginia Phillips, Guojun Bu, Dennis W. Dickson, Leonard Petrucelli, John D. Fryer

**Affiliations:** 1 Department of Neuroscience, Mayo Clinic Jacksonville, 4500 San Pablo Road, Jacksonville, FL 32224, USA; 2 Neurobiology of Disease Graduate Program, Mayo Graduate School, Jacksonville, FL 4500 San Pablo Road, Jacksonville, FL 32224, USA

## Abstract

Aberrant tau protein accumulation drives neurofibrillary tangle (NFT) formation in several neurodegenerative diseases. Currently, efforts to elucidate pathogenic mechanisms and assess the efficacy of therapeutic targets are limited by constraints of existing models of tauopathy. In order to generate a more versatile mouse model of tauopathy, somatic brain transgenesis was utilized to deliver adeno-associated virus serotype 1 (AAV1) encoding human mutant P301L-tau compared with GFP control. At 6 months of age, we observed widespread human tau expression with concomitant accumulation of hyperphosphorylated and abnormally folded proteinase K resistant tau. However, no overt neuronal loss was observed, though significant abnormalities were noted in the postsynaptic scaffolding protein PSD95. Neurofibrillary pathology was also detected with Gallyas silver stain and Thioflavin-S, and electron microscopy revealed the deposition of closely packed filaments. In addition to classic markers of tauopathy, significant neuroinflammation and extensive gliosis were detected in AAV1-Tau^P301L^ mice. This model also recapitulates the behavioral phenotype characteristic of mouse models of tauopathy, including abnormalities in exploration, anxiety, and learning and memory. These findings indicate that biochemical and neuropathological hallmarks of tauopathies are accurately conserved and are independent of cell death in this novel AAV-based model of tauopathy, which offers exceptional versatility and speed in comparison with existing transgenic models. Therefore, we anticipate this approach will facilitate the identification and validation of genetic modifiers of disease, as well as accelerate preclinical assessment of potential therapeutic targets.

## Introduction

Abnormal deposition of the tau protein is the hallmark feature of tauopathies, which encompasses a growing list of neurodegenerative diseases, including Alzheimer's disease (AD), frontotemporal dementia (FTD), progressive supranuclear palsy, corticobasal degeneration (CBD) and chronic traumatic encephalopathy (CTE). Additionally, pathogenic mutations in the *MAPT* gene encoding the tau protein are associated with FTD and parkinsonism linked to chromosome 17 (FTDP-17) (1–3) and CBD (4), indicating that tau dysfunction alone is sufficient to cause disease. Although not classified as a tauopathy, genetic variation at the tau locus has also been identified as a risk factor for Parkinson's disease (PD) (5), with varying degrees of tau pathology observed in PD and PD-related disorders including PD with dementia and dementia with Lewy bodies (6–13). Collectively, these findings indicate that a versatile model of tauopathy to explore the impact of different genetic *MAPT* coding variants, elucidate the role of tau in neurodegeneration and evaluate genetic modifiers of disease would greatly benefit the study of a wide range of conditions.

Despite the current availability of a number of transgenic mouse models of tauopathy, the necessity to control genetic background requires time-consuming breeding strategies to cross to other transgenic or knockout mice. Furthermore, the inflexible nature of the transgene prohibits the introduction of new tau mutations without the generation of an entirely new transgenic line. To address these limitations, we have developed a novel mouse model in which adeno-associated virus serotype 1 (AAV1) was used to express the FTD-associated P301L human tau protein (AAV1-Tau^P301L^) or control virus expressing GFP (AAV1-GFP) in C57BL/6 mice. At 6 months of age, widespread expression of human tau was found in AAV1-Tau^P301L^ mice, leading to significant accumulation of abnormally hyperphosphorylated tau species. Tau pathology was also detected with the conformational-dependent epitopes MC1 and Ab39, in addition to ubiquitin, Gallyas silver and Thioflavin-S staining. Electron microscopy (EM) revealed the deposition of straight filaments within both the cell soma and cellular processes of affected neurons. An additional feature of this model was neuroinflammation, with prominent microgliosis and astrocytosis. Importantly, while pathological changes were not associated with overt neuronal loss, the aberrant deposition of cleaved PSD95, a major postsynaptic scaffolding protein, is suggestive of significant structural changes within the synapse that may contribute to the behavioral abnormalities in exploration, anxiety, as well as learning and memory. These results indicate that the AAV1-Tau^P301L^ model recapitulates biochemical and histological hallmarks, as well as neuroinflammation and behavioral deficits characteristic of tauopathy but that these effects occur independently of neuronal cell death.

## Results

### Widespread expression of human tau in mice injected with AAV1-Tau^P301L^

**Figure 1.**
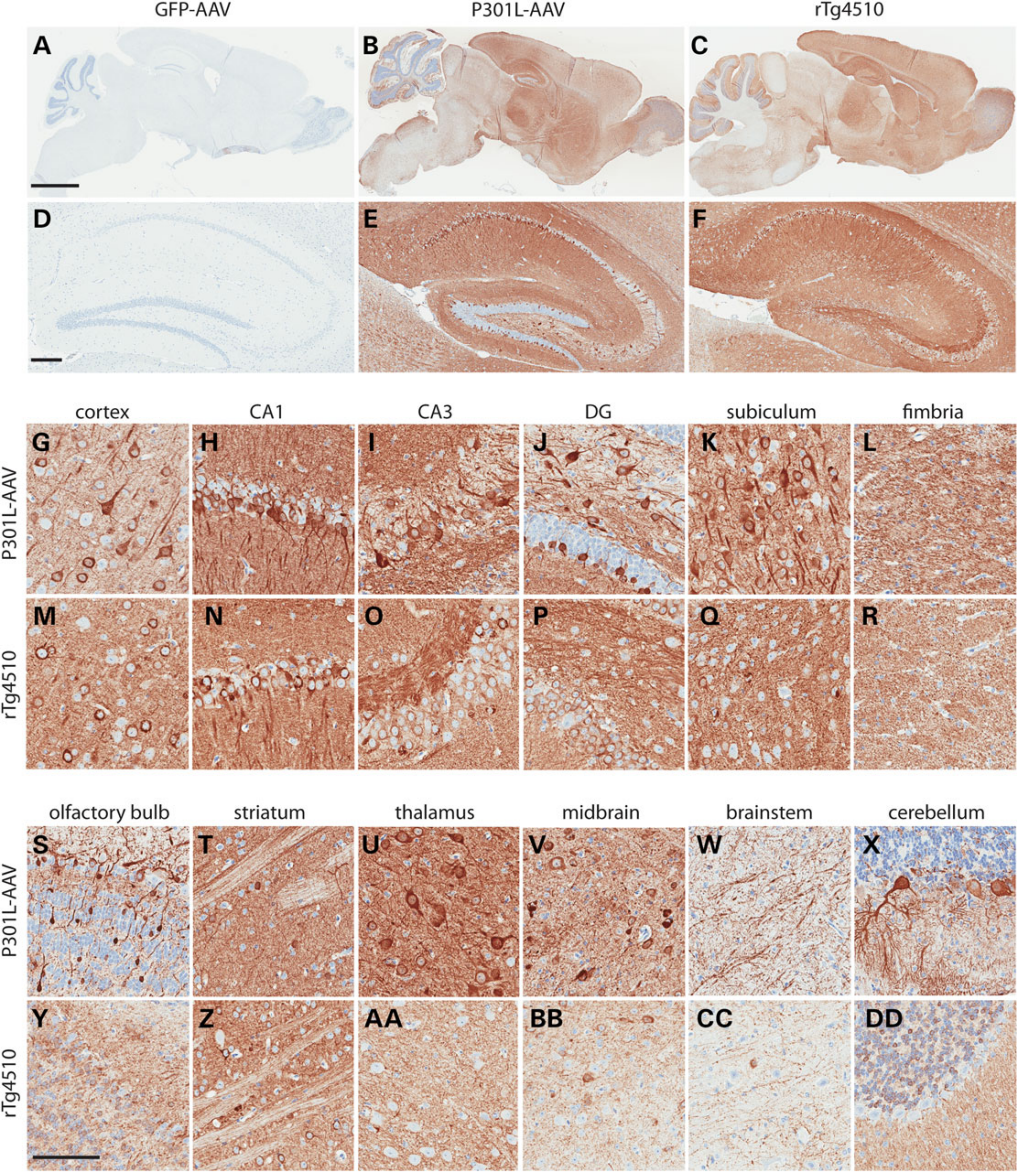
Widespread expression of human tau in AAV1-Tau^P301L^ model of tauopathy. Human tau-specific antibody (E1) reveals high level of expression throughout the brain and hippocampus in AAV1-Tau^P301L^-injected (**b** and **e**) and rTg4510 mice (**c** and **f**), whereas AAV1-GFP-injected mice are negative (**a** and **d**). AAV1-Tau^P301L^ is also highly expressed throughout the hippocampal network (**g–l**), as well as other regions of the brain (**s–x**). The pattern of human tau expression in the hippocampal network (**m–r**) and other regions of the brain (**y-dd**) is shown in comparison with rTg4510 model. Scale bar in a–c equals 2 mm; scale bar in d–f equals 200 µm; scale bar in g–dd equals 100 µm.

To assess the ability to model tauopathy with somatic brain transgenesis with AAV1-Tau^P301L^ on postnatal day 0, mice were harvested at 6 months of age and the level and distribution of human tau expression evaluated histologically (Fig. 1). Providing a point of reference for the pattern of expression, the level of human tau expression in various brain regions was compared with the commonly utilized rTg4510 mouse tauopathy model (14). As shown in Figure 1, similar to the rTg4510 model (Fig. 1c, f, m–r), a high level of human tau expression was observed in cortical and hippocampal regions in the AAV1-Tau^P301L^ model (Fig. 1b, e, g–l). Further increasing the utility of this model, human tau was also highly expressed in other areas of the brain including thalamic and midbrain regions (Fig. 1s–x), enabling the use of the AAV1-Tau^P301L^ model to evaluate genetic modifiers of non-cortical tauopathies. In addition, we evaluated human tau expression biochemically to provide an indication of the level of variability, and we observed minimal variation in human tau levels throughout the AAV1-Tau^P301L^ cohort (Supplementary Material, Fig. S1a). We also measured human tau levels by a quantitative immunoassay, which demonstrated that the average level of human tau in AAV1-Tau^P301L^ mice was 2.37 ± 0.087 ng/µg of brain tissue (mean ± SEM), which is significantly lower than rTg4510 mice that express ∼3.735 ± 0.057 ng/µg of human tau in the brain (Supplementary Material, Fig. S1b).

### Several markers of neurofibrillary pathology are detected in an AAV model of tauopathy

**Figure 2.**
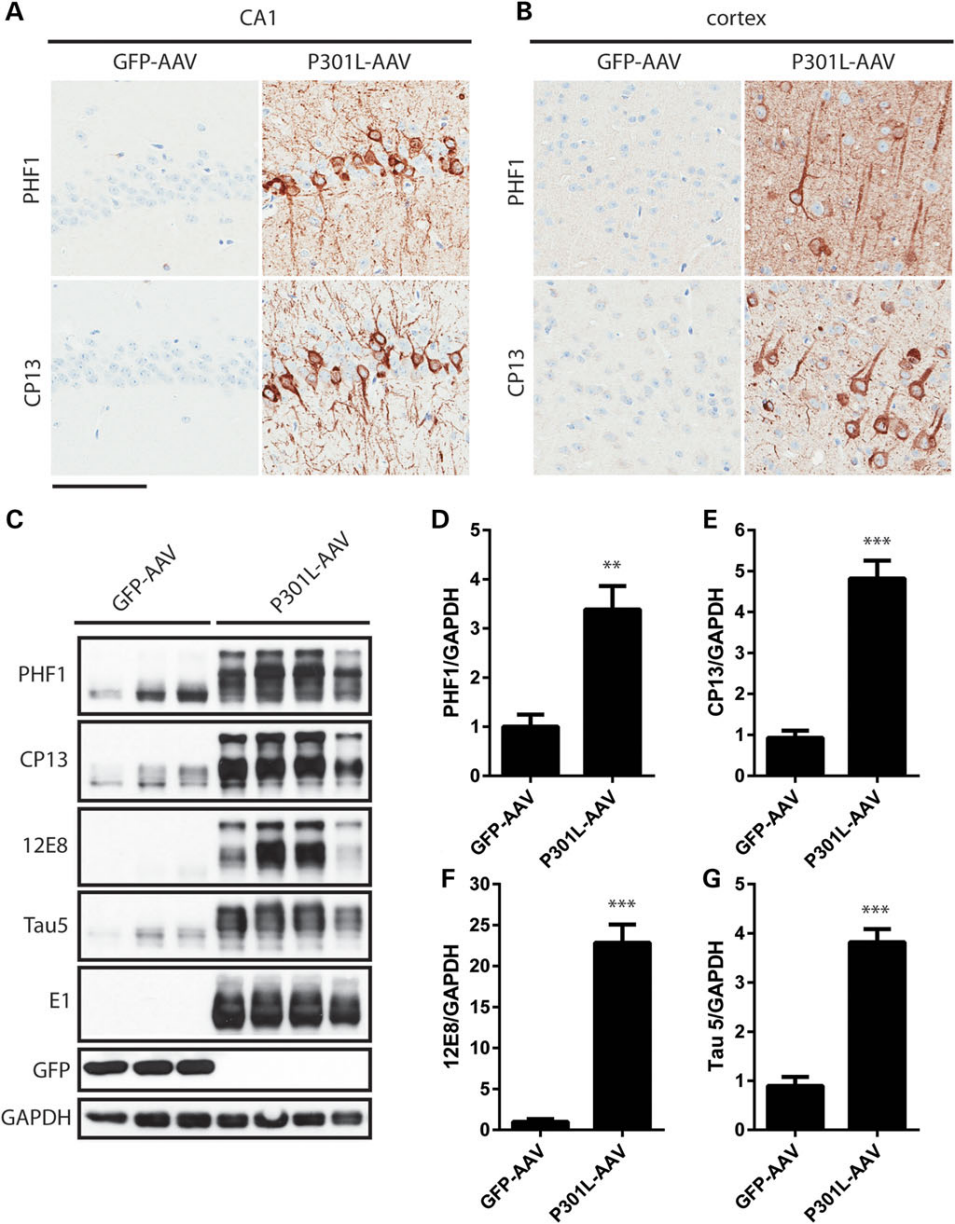
Accumulation of hyperphosphorylated tau species in AAV-driven model of tauopathy. (**a** and **b**) Representative images depicting the deposition of PHF1- and CP13-positive tau species in the CA1 field of the hippocampus (a), as well as the cortex (b). (**c**) Mice injected with AAV1-Tau^P301L^ express 4-fold higher tau levels in the forebrain as assessed by Tau 5 (recognizing both mouse and human tau), leading to accumulation of tau that is hyperphosphorylated on multiple epitopes. (**d**) Quantification of PHF1 normalized to GAPDH (*t* = 3.46, *P* = 0.0018); (**e**) CP13 (*t* = 6.26, *P* < 0.0001); (**f**) 12E8 (*t* = 7, *P* < 0.0001); (**g**) Tau 5 (*t* = 7.45, *P* < 0.0001). Scale bar is equal to 100 µm.

Given that neurofibrillary tangles (NFTs) contain tau species that are abnormally hyperphosphorylated on multiple epitopes, we wanted to determine the degree of tau hyperphosphorylation as well as the level of tau overexpression relative to endogenous tau in the AAV1-Tau^P301L^ model (Fig. 2). Strong immunoreactivity for the phospho-tau-specific antibodies PHF1 (pS396/404) and CP13 (pS202) was observed in hippocampal (Fig. 2a) and cortical (Fig. 2b) regions, in addition to other brain regions (data not shown), indicative of pathological changes in the tau protein. Total tau levels were evaluated biochemically using Tau 5 (Fig. 2c and g), which detects both mouse and human tau independently of phosphorylation state. This analysis revealed that while total tau levels were elevated by approximately 4-fold in AAV1-Tau^P301L^ mice (Fig. 2g), the abnormal phosphorylation of tau at the 12E8 epitope (pS262/356) was increased by more than 20-fold (Fig. 2c and f), most likely due to the very low level of phosphorylation at this pathologically relevant epitope under normal conditions.

**Figure 3.**
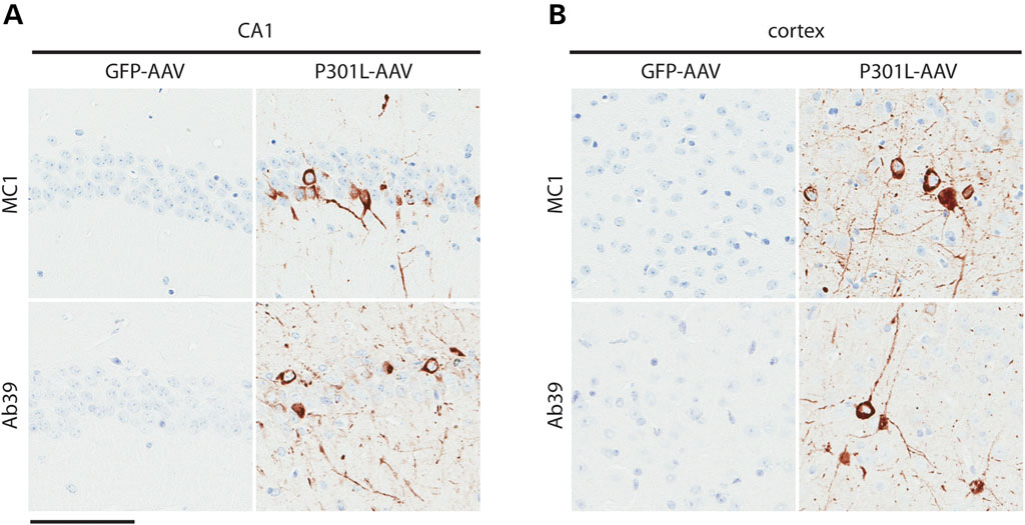
Deposition of abnormally folded and protease-resistant tau. (**a** and **b**) Injection of AAV1-Tau^P301L^ leads to the accumulation of tau positive for MC1, a well-characterized marker of abnormally folded tau, as well as proteinase K-resistant tau that is positive for Ab39, a marker of mature NFTs. Representative images from the CA1 field of the hippocampus (a) and cortex (b). Scale bar is equal to 100 µm.

**Figure 4.**
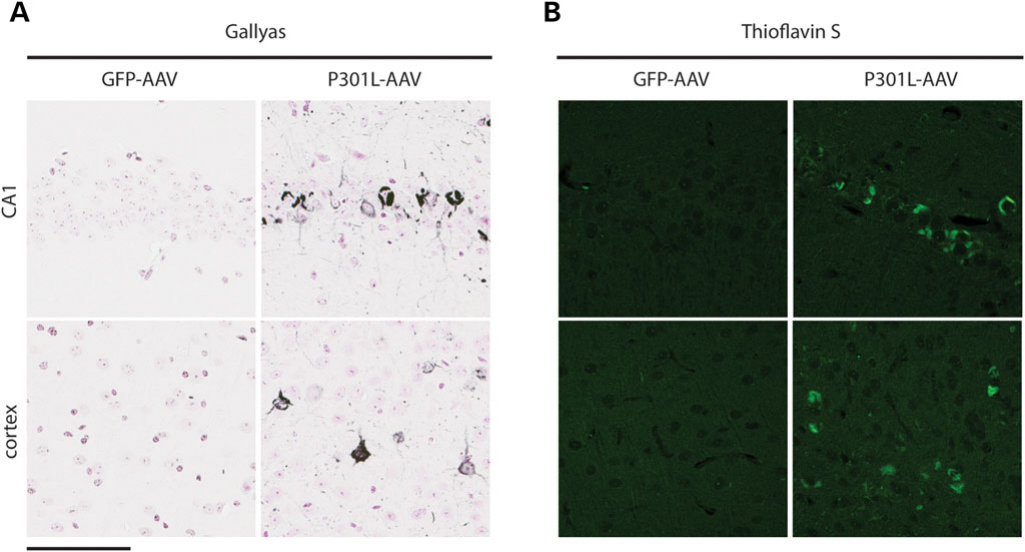
Thioflavin and Gallyas-positive tau deposition. (**a** and **b**) Expression of P301L-tau but not GFP leads to accumulation of insoluble tau species that are positive for Gallyas silver stain (a) and Thioflavin S (b) in CA1 and cortex. Scale bar is equal to 100 µm.

In order to further characterize the pathology in AAV1-Tau^P301L^-injected animals, we evaluated immunoreactivity for well-known markers of abnormal conformational changes in the tau protein. The MC1 antibody, which detects a conformational change that occurs early in NFT formation (15,16), labeled inclusions in the hippocampus and cortex (Fig. 3a and b) as well as other brain regions of AAV1-Tau^P301L^ mice (data not shown). To detect mature NFT pathology, we pretreated tissue sections with proteinase K and subsequently stained with the conformational-dependent antibody Ab39 (17–19). The strong positivity for Ab39 in hippocampal and cortical regions (Fig. 3a and b) was indicative of the formation of a mature, protease-resistant form of neurofibrillary pathology. In addition, inclusions in AAV1-Tau^P301L^ mice were also positive on Gallyas silver stain and Thioflavin S fluorescent microscopy (Fig. 4), denoting argyrophilic lesions (20) and the adoption of β-pleated sheet structure (21), respectively. These findings suggest that a wide range of pathology at varying stages of maturity is observed in the AAV1-Tau^P301L^ model, which models changes observed in human tauopathies. We also quantified the number of NeuN-positive nuclei (Supplementary Material, Fig. S2) but detected no significant difference between AAV1-GFP and AAV1-Tau^P301L^ mice (*t* = 1.4, *P* = 0.2), indicating that the deposition of pathological forms of tau was not accompanied by significant neuronal loss in this model at 6 months of age.

As pathological tau species purified from AD brain have also been shown to be ubiquitinated (22), and ubiquitin is present within NFTs (23), we evaluated whether ubiquitin-positive lesions were present in AAV1-Tau^P301L^ mice. Remarkably, ubiquitinated inclusions are observed in mice injected with AAV1-Tau^P301L^, but not in control animals injected with AAV1-GFP (Supplementary Material, Fig. S3), verifying that AAV1 infection and/or GFP overexpression alone are not sufficient to drive the deposition of ubiquitin within aggregates.

### Tau filaments detected at the ultrastructural level

**Figure 5.**
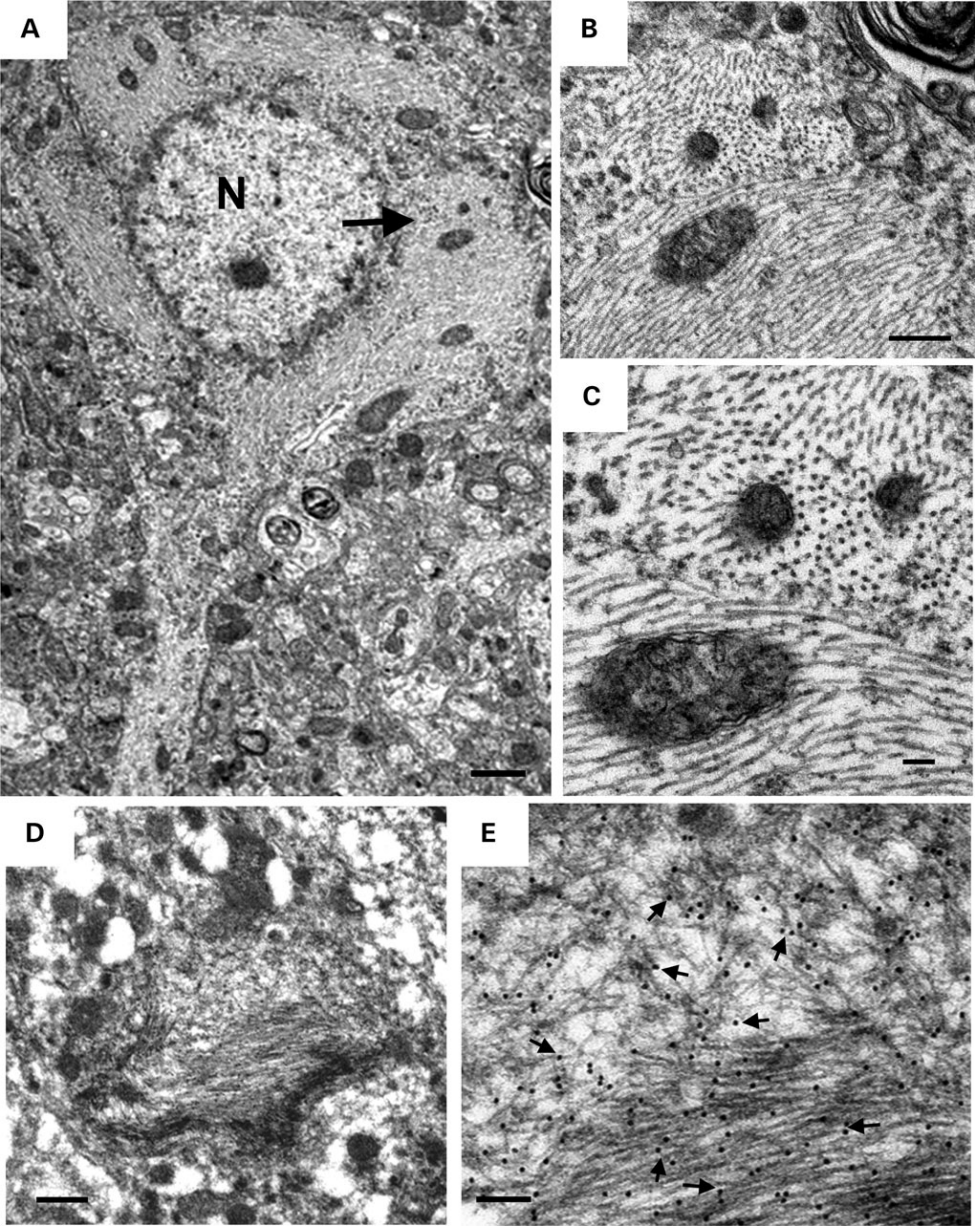
Tau filament formation in AAV1-Tau^P301L^ model of tauopathy. (**a**) Electron micrograph of a cortical neuron containing bundles of packed filaments that filled almost the entire cell body (N, nucleus). Arrow points to enlargement in (**b**) that shows the inclusion is not membrane-bound. (**c**) Further enlargement of (b) shows longitudinal, oblique and cross sections of filaments with diameters of 11–17 nm. Most of the filaments are straight. Some cross sections have a central lumen. Note the close proximity of filaments and mitochondria. (**d**) A filamentous aggregate excludes cytoplasmic organelles to its periphery. (**e**) Enlargement of the aggregate shows packed and loose filaments heavily labeled with E1 antibody that is specific for human tau. Scale bars are equal to 1 µm (a); 0.2 µm (b); 0.25 µm (d); 50 nm (c and e).

**Figure 6.**
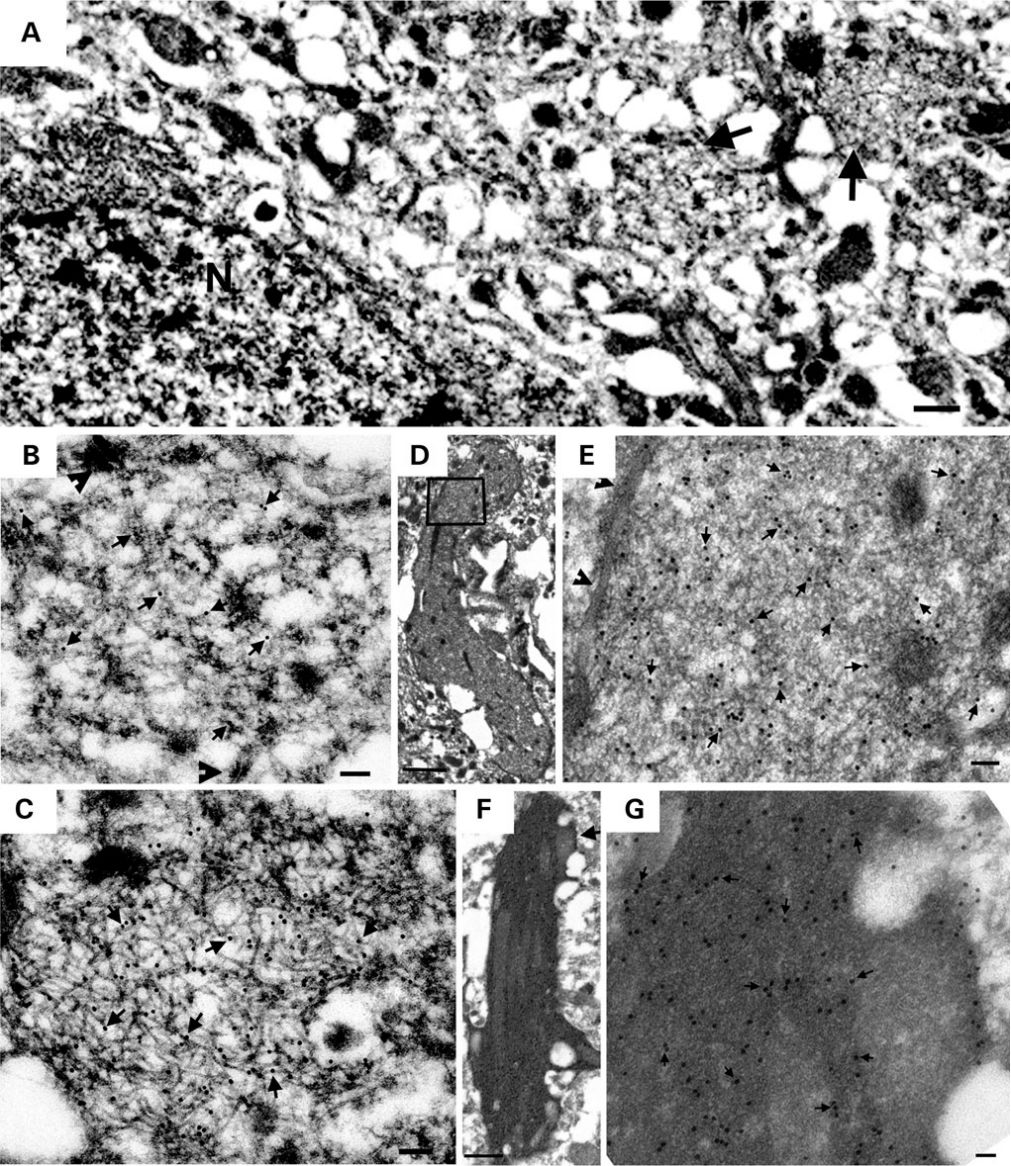
AAV1-Tau^P301L^ drives tau filament formation in neuronal processes. (**a**) A dentate granule cell (N) and two nearby neurites (arrows). (**b** and **c**) Enlargements of neurites in (a) show moderate labeling on less well-formed filaments (b) and heavy labeling of E1 on well-formed filaments (c). Arrows point to 18-nm gold particles. Arrowheads point to synapses. (**d**) A swollen, thinly myelinated neurite filled with E1-positive filaments. (**e**) Boxed area in (d) shows randomly orientated filaments. Arrowheads point to myelin sheathes. (**f**) A dense dystrophic neurite filled with E1 immunoreactivity. Arrowed area is enlarged in (**g**) showing many gold particles (arrows). Arrows point to gold particles. Scale bars are equal to 0.5 µm (a); 50 nm (b and c); 1 µm (d); 100 nm (e); 0.5 µm (f); 50 nm (g).

Ultrastructural analysis of the AAV1-Tau^P301L^ model showed tau filaments in neuronal cytoplasmic inclusions that were not membrane-bound, as well as in both myelinated and unmyelinated cell processes (Figs. 5 and 6). Filaments were predominantly straight, with diameters of 11–17 nm (Fig. 5). A lumen was visible in some filaments in cross section, suggesting a tubule-like morphology. The packed filaments were oriented parallel to each other, and they excluded cytoplasmic organelles with the exception of mitochondria, which seemed to be aligned with and positioned in the same orientation as the filaments (Fig. 5a–c). Immuno-EM with an antibody specific for human tau (E1) verified these filaments were composed of human tau (Fig. 5d and e). Also noted was a range of deposition patterns, such as filamentous aggregates containing both tightly packed and loose filaments (Fig. 5d and e), or non-filamentous tau based upon strong immunolabeling with E1 antibody that was diffusely distributed throughout the cytoplasm, which may represent ‘pre-tangle’ pathology (Supplementary Material, Fig. S4a and b). E1 immunoreactivity was also detected in myelinated and unmyelinated cell processes, with the maturity of tau aggregates ranging from less-organized to well-formed filaments (Fig. 6a–e). Dystrophic neurites filled with dense, tightly compacted tau were also observed (Fig. 6f and g). Therefore, a very extensive range of pathology was detected in both the cell soma and processes in the AAV1-Tau^P301L^ model similar to that observed in human tauopathies.

### Tau pathology is associated with prominent gliosis and inflammatory changes

**Figure 7.**
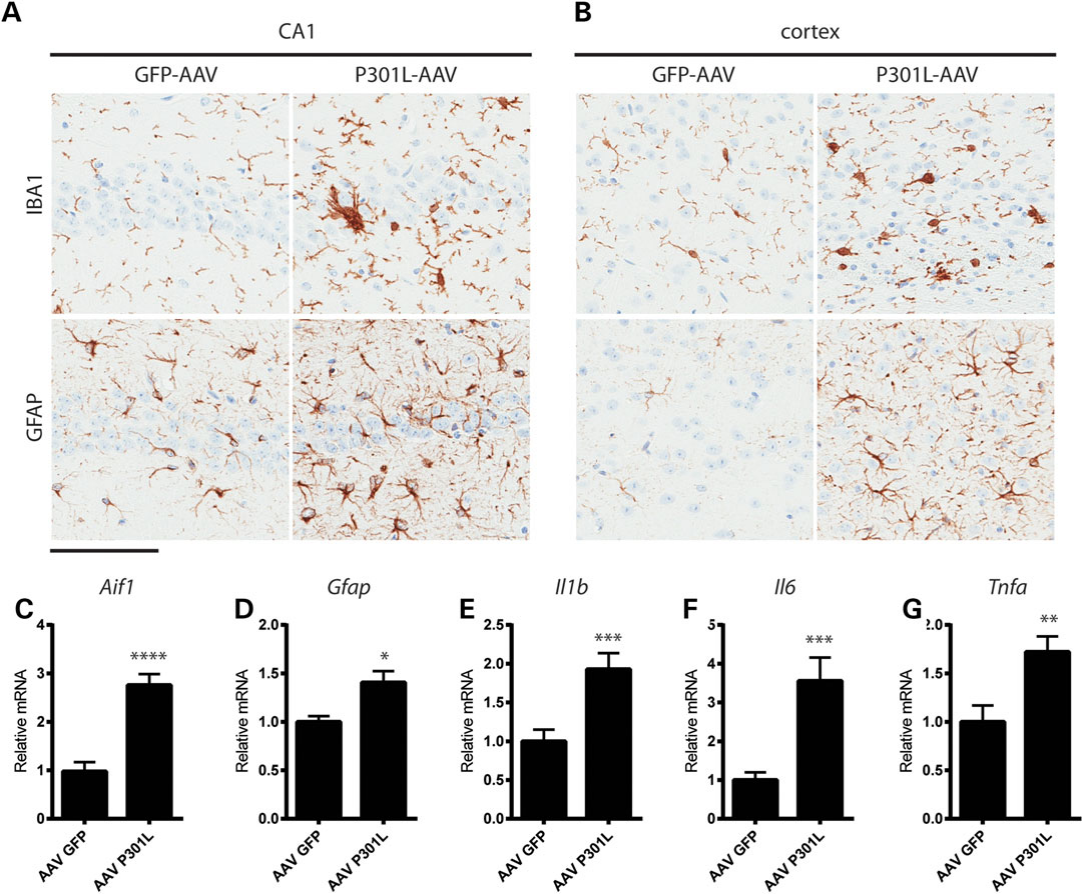
Tau pathology is associated with robust gliosis and inflammation. (**a** and **b**) Microgliosis and astrocytosis detected by Iba1 and GFAP, respectively, in the CA1 field of the hippocampus (a) and cortical regions (b). (**c**–**g**) Inflammatory markers were evaluated by RT-qPCR. (c) *Aif1* (*t* = 5.3, *P* < 0.0001), (d) *Gfap* (*t* = 2.5, *P* = 0.017), (e) *Il1b* (*t* = 3.15, *P* = 0.004), (f) *Il6* (*t* = 3.17, *P* = 0.004) and (g) *Tnfa* (*t* = 2.9, *P* = 0.007) were all significantly elevated in AAV1-Tau^P301L^ mice compared with AAV1-GFP controls. Scale bar is equal to 100 µm *P < 0.05, **P < 0.01, ***P < 0.005, ****P < 0.0001.

Several lines of evidence indicate that increasing inflammation exacerbates tau pathology (24–27). Moreover, tau pathology is associated with inflammation in both human tissue (28–30) and animal models (24,31,32), suggesting that a toxic interplay between neuroinflammation and tau pathology regulates disease progression. To evaluate whether inflammation was observed in animals injected with AAV1-Tau^P301L^, we first examined glial activation histologically. The intensity of immunolabeling in addition to the morphology of IBA1-positive microglia was markedly altered in the hippocampus and cortex of mice injected with AAV1-Tau^P301L^, indicative of microglial activation (Fig. 7a and b). Elevated expression and altered morphology of GFAP-positive astrocytes was also noted in the same brain regions (Fig. 7a and b), demonstrating that the presence of tau pathology is associated with both microgliosis and astrocytosis in this model. To further investigate the inflammatory changes driven by AAV1-Tau^P301L^ expression, we utilized RT-qPCR to measure mRNA levels and identified a significant upregulation in *Aif1* (encoding Iba1) and *Gfap* transcripts, as well as the pro-inflammatory cytokines *IL-1β*, *IL-6* and *TNF-α* (Fig. 7c–g). Intriguingly, these same pro-inflammatory cytokines have also been reported to be elevated in plasma, cerebrospinal fluid and brain from patients with AD or mild cognitive impairment (33–39), further supporting the pathological relevance of the AAV1-Tau^P301L^ model.

### Expression of AAV1-Tau^P301L^ leads to behavioral abnormalities characteristic of tauopathy

**Figure 8.**
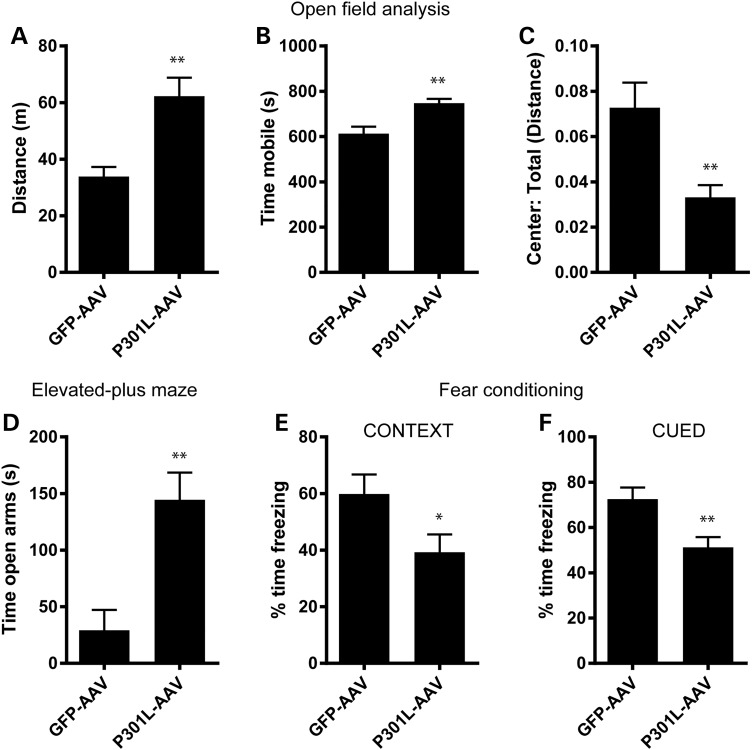
Behavioral abnormalities in AAV1-Tau^P301L^ model of tauopathy. (**a**–**c**) Behavior in the OFA revealed that AAV1-Tau^P301L^ mice exhibited an increase in total distance traveled (a; *t* = 3.1, *P* = 0.004) and time spent mobile (b; *t* = 3.9, *P* = 0.0006), and a decrease in the ratio of distance traveled in the center of the box compared with the total distance traveled (c; *t* = 3.6, *P* = 0.001). (**d**) Exploratory behavior was also evaluated in the EPM, which detected a significant increase in the time spent in open arms (*t* = 3.3, *P* = 0.0025). (**e** and **f**) Contextual fear conditioning was utilized to measure learning and memory. AAV1-Tau^P301L^ mice were significantly impaired in both contextual (e; *t* = 2.1, *P* = 0.04) and cued (f; *t* = 3, *P* = 0.005) versions of the task, indicative of hippocampal and amygdala-dependent learning and memory deficits. *P < 0.05, **P ≤ 0.005.

**Figure 9.**
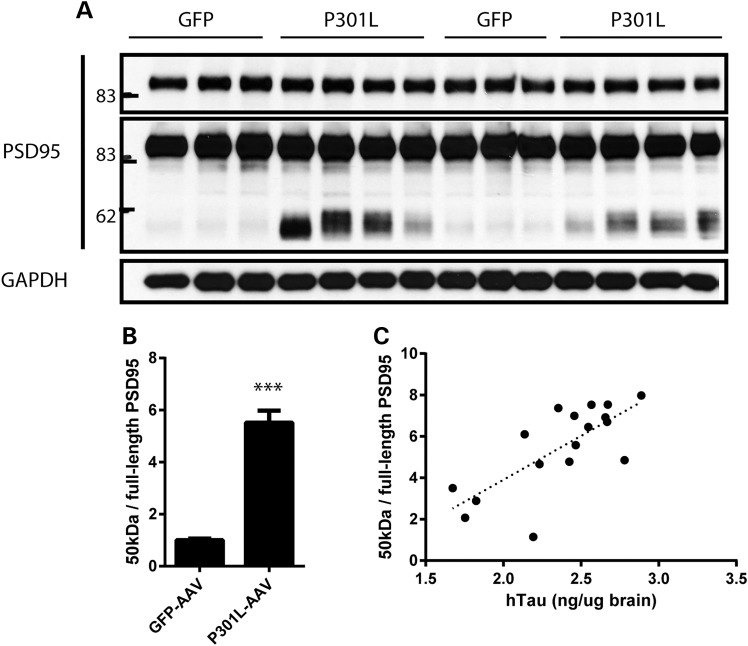
Aberrant proteolysis of PSD-95 in AAV1-Tau^P301L^ mice. (**a**) Accumulation of a 50-kDa PSD95 fragment was observed in mice injected with AAV1-Tau^P301L^, whereas the levels of full-length PSD95 were unchanged. (**b**) Quantification of the 50-kDa fragment normalized to full-length PSD95 revealed a highly significant increase in AAV1-Tau^P301L^ mice (*t* = 7.47, *P* < 0.0001). (**c**) The 50-kDa/full-length PSD95 ratio is significantly correlated with the levels of human tau in animals injected with AAV1-Tau^P301L^ (*r* = 0.74, *P* = 0.0008). ***P < 0.0001.

To determine the behavioral impact of AAV1-Tau^P301L^-driven pathology, we evaluated performance on tasks designed to assess exploration, anxiety, as well as learning and memory that are often characteristically abnormal in various human tauopathies. In the open-field assay (OFA), AAV1-Tau^P301L^ mice exhibited hyperactivity as assessed by total distance traveled and time spent mobile (Fig. 8a and b). In addition to hyperactivity, AAV1-Tau^P301L^ mice also displayed a decreased tendency to explore the center of the open field (Fig. 8c), which is typically characteristic of increased anxiety. However, in the elevated plus maze (EPM), AAV1-Tau^P301L^ mice actually spent a greater amount of time in the open arms (Fig. 8d), indicating that this model is characterized by aberrant exploratory behavior and disinhibition, similar to the results obtained in the rTg4510 transgenic mouse model (40). AAV1-Tau^P301L^ mice also exhibited deficits in a contextual fear conditioning paradigm, with significant memory impairments in their ability to associate either the context (Fig. 8e) or an auditory cue (Fig. 8f), reflective of hippocampal and amygdala dysfunction. As similar behavioral abnormalities have been noted in rTg4510 mice (40), as well as other models of tauopathy (41–46), the utilization of somatic brain transgenesis to deliver AAV1-Tau^P301L^ to neonatal mice recapitulates several key features characteristic of tauopathy but in the absence of overt neuronal loss. Therefore, to determine whether synaptic abnormalities were present in AAV1-Tau^P301L^ mice, we evaluated the expression of the postsynaptic scaffolding protein, PSD95. While the levels of full-length PSD95 were unaltered (Fig. 9a, *t* = 0.41, *P* = 0.69), we observed the appearance and significant accumulation of a 50-kDa fragment in all AAV1-Tau^P301L^ mice (Fig. 9a and b, *t* = 7.47, *P* < 0.0001), a fragment that has previously been reported to be generated by calpain under conditions of excitotoxicity and ischemia/reperfusion injury (47,48). We also confirmed that human tau expression was positively correlated with the levels of the 50-kDa PSD95 fragment (Fig. 9c, *r* = 0.74, *P* = 0.0008), suggesting that the aberrant processing of PSD95 is a result of AAV1-Tau^P301L^ expression.

## Discussion

We demonstrate that widespread expression of human tau was detected in mice injected with AAV1-Tau^P301L^, leading to the accumulation and deposition of hyperphosphorylated and abnormally folded tau species in NFTs and neuritic inclusions, which are also detected with Gallyas silver stain and Thioflavin S. Perhaps most importantly, the tau pathology produced by the expression of AAV1-Tau^P301L^ recapitulates human tauopathies at the ultrastructural level as indicated by the presence of pre-tangles, mature neurofibrillary tangles, neuropil threads and dystrophic neurites. In addition to tau pathology, there was prominent gliosis, behavioral changes and synaptic abnormalities. Collectively, the data support the use of this model in preclinical assessment of tau-based therapies. Moreover, given the very extensive range of pathology that is observed in both the cell soma and processes, this model offers the capability to evaluate the efficacy of therapeutic strategies at modulating both early- and late-stage pathologies within the same animal. The fact that tau filaments are not detected within neuronal processes in the rTg4510 model (49), which is presumably the result of a very high level of tau expression driving rapid aggregation in the cell soma, highlights an additional advantage of the AAV1-Tau^P301L^ model. With the ease of delivery and the liberation from constraints of complex genetic backgrounds, we anticipate this approach will also rapidly accelerate the identification and validation of genetic modifiers of disease, such as evaluating whether overexpression or deletion of a gene of interest impacts the development or progression of tauopathy.

The fact that tau pathology and tauopathy-associated behavioral abnormalities appear to occur independently of neuronal loss in our AAV-based model is in agreement with reports in other tau models, including the rTg4510 and humanized tau mouse models (50,51), although future studies will need to assess whether neuronal loss is observed in the AAV1-Tau^P301L^ model beyond 6 months of age. Of note, electrophysiological changes and structural atrophy of dendritic spines have been shown to precede significant deposition of neurofibrillary pathology and neuronal loss in rTg4510 mice (52,53), which suggests that synaptic dysfunction mediated by pathological forms of tau is the basis for the observed behavioral deficits, rather than neuronal loss. Our current discovery that cleaved PSD95 accumulates in AAV1-Tau^P301L^ mice may now provide novel insight into the mechanism by which tau pathology promotes structural synaptic remodeling. The finding that PSD95 exhibits an aberrant redistribution from the synapse to the cell soma prior to the appearance of neuronal loss in the JNPL3 mouse model of tauopathy (54) is yet additional support for a key role of PSD95 abnormalities in driving tau-mediated neuronal dysfunction. Although cleavage of PSD95 does not appear to have been evaluated in JNPL3 mice, it is possible the abnormal localization of PSD95 to the cell soma is a consequence of increased proteolytic processing, similar to the AAV1-Tau^P301L^ model. In addition, it is also possible cleaved PSD95 functions in a dominant-negative manner, binding to synaptic proteins and downstream signaling molecules but lacking the critical domains required to associate with the cytoskeleton in the postsynaptic density. This could result in the dissociation of synaptic activity from prosurvival and neurotrophic signaling cascades, similar to the proposed consequence of PSD95 cleavage in cerebral ischemia (47). Thus, additional studies are needed to investigate the functional role of cleaved PSD95 in synaptic activity, as well as to determine whether the cleavage of PSD95 in AAV1-Tau^P301L^ animals occurs in a calpain and calcium-dependent manner. These studies are critical given the recent demonstration that increased expression of the endogenous calpain inhibitor calpastatin dramatically slows disease progression in JNPL3 mice (55), which may identify an effective therapeutic strategy to prevent PSD95 cleavage and block tau-mediated synaptic dysfunction.

Somatic brain transgenesis could also be utilized to compare the resulting phenotype with different tau mutations, such as the V75A mutation for which pathogenicity remains undetermined (56), or the A152T variant which appears to modulate risk for AD and the spectrum of FTD disorders (57,58). In addition, although the A239T tau variant is reported to be benign (59,60), a recent study identified this mutation in a patient carrying a repeat expansion in the *C9ORF72* gene (61), which is the most common cause of amyotrophic lateral sclerosis (ALS) and frontotemporal lobar degeneration (FTLD) (62,63). This patient was diagnosed with FTLD, and following autopsy and the subsequent neuropathological assessment, the case was determined to be a tauopathy with mixed TDP-43 and p62 pathology (61). Siblings that carried the *C9ORF72* repeat expansion but not the A239T tau mutation developed ALS with no cognitive symptoms (61), suggesting that the presence of an otherwise benign tau variant shifted the clinical presentation from ALS to FTLD. These findings, in addition to the discovery that FTLD patients carrying the *C9ORF72* expansion exhibit more extensive tau pathology than FTLD caused by mutations in progranulin (64), may also indicate that the *C9ORF72* mutation impacts tau accumulation. As animal models of the *C9ORF72* repeat expansion begin to emerge (65), our AAV-based model of tauopathy provides the capability to explore this very intriguing relationship between *C9ORF72* and tau pathology *in vivo*.

Finally, as the *C9ORF72* repeat expansion is able to drive aggregation of a mutant form of tau that was previously considered non-pathogenic, it is possible that other tau mutations previously deemed benign owing to their identification in control patients may similarly modulate risk for neurodegeneration. While repetitive head injury is considered to be the cause of tau pathology seen in CTE (66,67), given the rarity of this disorder it is clear additional factors must contribute, including genetic predisposition. Perhaps tau variants considered non-pathogenic are not sufficient to cause tauopathy but alter the sensitivity and/or threshold for disease. Therefore, somatic brain transgenesis also offers the means to evaluate whether the pathogenicity of tau mutations can be altered in the presence of an additional insult, such as *C9ORF72* mutations or repetitive head trauma.

## Materials and Methods

### Antibodies

PHF1 (pS396/S404), CP13 (pS202) and MC1 (conformational epitope) were kindly provided by Dr Peter Davies (Feinstein Institute for Medical Research, North Shore LIJ Health Care System). Ab39 (conformational epitope) was provided by Dr Shu-Hui Yen (Mayo Clinic, Jacksonville, FL) (19,68). 12E8 (pS262/S356) was provided by Dr Peter Seubert (previously at Elan Pharmaceuticals, San Francisco, CA). Tau 5 (total mouse and human tau) was provided by our late and dear colleague Dr Skip Binder (Northwestern University Medical School, Chicago, IL). E1 (human-specific tau antibody) was generated by our group against amino acid residues 19–33 within exon 1 of human tau (69–71). In addition, we purchased anti-GFP from Life Technologies (A11122, Grand Island, NY), anti-ubiquitin was purchased from Millipore (Ubi-1, Billerica, MA), anti-IBA1 was purchased from Wako Chemicals (019–19741, Richmond, VA), anti-GFAP was purchased from Biogenex (PU020-UP, Fremont, CA), anti-PSD95 was purchased from Abcam (ab18258, Cambridge, MA), and anti-GAPDH was purchased from Meridian Life Science, Inc. (Memphis, TN). Secondary antibodies were obtained from Jackson ImmunoResearch Laboratories, Inc. (West Grove, PA).

### Construct generation and viral production

V5-tagged Tau^P301L^ or GFP expression plasmids were cloned into an AAV vector. The constructs were sequence-verified using ABI3730 with Big Dye chemistry following manufacturer's protocols (Applied Biosystems, Foster City, CA, USA). AAV1-Tau^P301L^ and AAV1-GFP were prepared by the following methods. AAV vectors expressing Tau^P301L^ or GFP under the control of the cytomegalovirus enhancer/chicken β-actin promoter, a woodchuck post-transcriptional regulatory element and the bovine growth hormone polyA were generated by plasmid transfection with AAV helper plasmids in HEK293T cells. Forty-eight hours after transfection, the cells were harvested and lysed in the presence of 0.5% sodium deoxycholate and 50 U/ml Benzonase (Sigma, St. Louis, MO) by freeze thawing, and the virus was isolated using a discontinuous iodixanol gradient. The genomic titer of each virus was determined by quantitative PCR.

### Intracerebroventricular injections

All animal procedures were approved by the Mayo Institutional Animal Care and Use Committee and are in accordance with the National Institutes of Health Guide for the Care and Use of Laboratory Animals (NIH Publications No. 80–23, revised 1996). AAV1-Tau^P301L^ and AAV1-GFP were injected intracerebroventricularly (ICV) in C57BL/6 mouse pups on postnatal day 0 (2.7E+10 viral particles/ventricle; 2 µl/ventricle). ICV injections were performed as described (72). Newborn mice were cryoanesthetized and subsequently placed on a cold metal plate. A 30-gauge needle was used to pierce the skull just posterior to bregma and 2 mm lateral to the midline, and 2 µl of AAV was injected into the lateral ventricles. At 6 months of age, behavioral testing was performed. Subsequently, animals were deeply anesthetized with sodium pentobarbital prior to transcardial perfusion with phosphate-buffered saline. The brain was removed and bisected along the midline. Half was drop-fixed in 10% neutral buffered formalin (Fisher Scientific, Waltham, MA) overnight at 4°C for histology, whereas the other half was frozen, after removal of the olfactory bulb and cerebellum, for biochemical studies.

### Preparation of brain homogenates and western blot analysis

Mouse hemi-brains were weighed and homogenized in 10× volume of buffer [50 mm Tris base (pH 8), 274 mm NaCl, 5 mm KCl, with 1× protease and phosphatase inhibitors (Thermo Scientific, Waltham, MA)]. The homogenate, after addition of 1% SDS final concentration, was sonicated and then centrifuged for 15 min at 16 000 g at 4°C to remove cellular debris. A BCA protein assay (Pierce Biotechnology, Rockford, IL) was performed on the supernatant. Protein (10 µg) from each sample was diluted in dH_2_O, 2× Tris–glycine SDS sample buffer (Life Technologies) and 5% beta-mercaptoethanol (Sigma-Aldrich) and heat-denatured for 5 min at 95°C. Samples were run on 10% or 4–20% SDS–PAGE Tris–glycine gels (Life Technologies) and transferred to PVDF membrane (Millipore). Membranes were blocked in 5% non-fat dry milk in TBS/0.1% Triton X-100 and incubated overnight in primary antibody diluted in 5% milk in TBS/0.1% Triton X-100 rocking at 4°C. Membranes were incubated in HRP-conjugated secondary antibodies (1:5000) for 1 h at room temperature and detected by ECL (PerkinElmer). Bands were quantified using Scion Image by analyzing pixel density, and protein levels were normalized to the protein loading control.

### RNA preparation and qRT-PCR

Total RNA was isolated from brain tissue using the Aurum Total RNA mini isolation kit (Biorad) according to manufacturer's instructions with in-column DNase I treatment. Random-primed reverse transcription was performed according to manufacturer protocols (Invitrogen—Life Technologies, Grand Island, NY). cDNA was added to a reaction mix (10 µl final volume) containing 300 nm gene-specific primers and Universal SYBR green supermix (Biorad, Hercules, CA). All samples were run in triplicate and were analyzed on an ABI 7900 HT Fast Real Time PCR instrument (Applied Biosystems—Life Technologies). Relative gene expression was normalized to GAPDH controls and assessed using the 2^−ΔΔCT^ method. Primer sequences are as follows (5′–3′): *Gapdh* F: CTGCACCACCAACTGCTTAG, *Gapdh* R: ACAGTCTTCTGGGTGGCA GT, *Aif1 (Iba1)* F: GGATTTGCAGGGAGGAAAAG *Aif1* (Iba1) R: TGGGATCATCGAGGAATTG, *Gfap* F: GGAGAGGGACAACTTTGCAC, *Gfap* R: AGCCTCAGGTTGGTTTCATC, *Il1b* F: CCTGCAGCTGGAGAGTGTGGAT, *Il1b* R: TGTGCTCTGCTTGTGAGGTGCT, *Il6* F: CAAAGCCAGAGTCCTTCAGAG, *Il6* R: AGGAGAGCATTGGAAATTGG, *Tnfa* F: AGCCCACGTCGTAGCAAACCAC, *Tnfa* R: AGGTACAACCCATCGGCTGGCA, *Tgfb* F: TGGAGCAACATGTGGAACTC, *Tgfb* R: GACAGCCACTCAGGCGTATC, *Bdnf* F: CAATGCCGAACTACCCAATC, *Bdnf* R: TGGTCAGTGTACATACACAGGAAG.

### Histology and immunohistochemistry

The half brain fixed in 10% formalin was embedded in paraffin wax, sectioned in a sagittal plane at 5 micron thickness and mounted on glass slides. The tissue sections were deparaffinized in xylene and rehydrated in a graded series of alcohols. Antigen retrieval was performed by steaming in distilled water for 30 min, and endogenous peroxidase activity was blocked by incubation in 0.03% hydrogen peroxide. Sections were then immunostained using the DAKO Autostainer (DAKO North America, Carpinteria, CA) and the DAKO EnVision+HRP system. The stained slides were then dehydrated, cover-slipped and scanned with the Aperio Slide Scanner (Aperio, Vista, CA). Gallyas silver stain and Thio-S staining were performed as previously described (73).

### Electron microscopy

The cortex and hippocampus from 6-month-old post-injected mice were processed for transmission EM as well as post-embedding immunogold EM as previously reported (74). Ultrathin sections were examined in a Philips 208S electron microscope fitted with a Gatan 831 Orius digital camera. Images were processed with Adobe Photoshop CS5 software.

### Behavioral analysis

A behavioral battery consisting of OFA, EPM test, and contextual and cued fear conditioning (CFC) was performed on consecutive days, as described (40). Mice were acclimated to the room of testing for 1 h, and all tests were performed during the first half of the light cycle, except for CFC. All behavioral equipment was extensively cleaned with 30% ethanol between each animal. Mice were returned to their home cage and homeroom after each test.

#### Open-field assay

Mice were placed in the center of an open-field arena (40 × 40 × 30 cm, W × L × H) and allowed to roam freely for 15 min. An overhead camera was used to track movement with AnyMaze software (Stoelting Co., Wood Dale, IL), and mice were analyzed for multiple measures, including total distance traveled, average speed, time mobile, and distance traveled in an imaginary ‘center’ zone (20 × 20 cm).

#### Elevated plus maze test

As a formal test of anxiety/exploration, the entire maze is elevated 50 cm from the floor and consists of four arms (50 × 10 cm) with two of the arms enclosed with roofless gray walls (35 × 15 cm, L × H). Mice were tested by placing them in the center of the maze facing an open arm, and their behavior was tracked for 5min with an overhead camera and AnyMaze software.

#### Contextual and CFC test

This test was conducted in a sound attenuating chamber with a grid floor capable of delivering an electric shock, and freezing was measured with an overhead camera and FreezeFrame software (Actimetrics, Wilmette, IL). Mice were initially placed into the chamber undisturbed for 2 min, during which time baseline freezing behavior was recorded. An 80-dB white noise served as the conditioned stimulus (CS) and was presented for 30 s. During the final 2 s of this noise, mice received a mild foot shock (0.5 mA), which served as the unconditioned stimulus (US). After 1 min, another CS–US pair was presented. The mouse was removed 30 s after the second CS–US pair and returned to its home cage. Twenty-four hours later, each mouse was returned to the test chamber and freezing behavior was recorded for 5 min (context test). Mice were returned to their home cage and placed in a different room than previously tested in reduced lighting conditions for a period of no less than 1 h. For the auditory CS test, environmental and contextual cues were changed by: wiping testing boxes with 30% isopropyl alcohol instead of 30% ethanol; replacing white house lights with red house lights; placing a colored plastic triangular insert in the chamber to alter its shape and spatial cues; covering the wire grid floor with opaque plastic and altering the smell in the chamber with vanilla extract. The animals were placed in the apparatus for 3 min and then the auditory CS was presented and freezing was recorded for another 3 min (cued test). Baseline freezing behavior obtained during training was subtracted from the context or cued tests to control for animal variability. No differences were observed in baseline freezing in this test were observed between groups.

### Statistical analyses

To determine whether differences between the AAV1-Tau^P301L^ (*n* = 19) and AAV1-GFP-injected (*n* = 11) animals were statistically significant, differences between the two groups were assessed using unpaired two-tailed *t* tests in GraphPad Prism. All data are presented as mean ± SEM. *P* < 0.05 was considered statistically significant.

## Supplementary Material

Supplementary Material is available at *HMG* online.

*Conflict of Interest statement.* None declared.

## Funding

This work was supported by Mayo Clinic Foundation (L.P. and J.D.F.), National Institutes of Health/National Institute on Aging [ADRC 2 P50 AG016574-16 (L.P.)], National Institutes of Health/National Institute of Neurological Disorders and Stroke [R01NS089544 (L.P.)] and BrightFocus Foundation A2013546S (L.P.). Funding to pay the Open Access publication charges for this article was provided by Mayo Clinic.

## Supplementary Material

Supplementary Data
