# Supplementary material for: Tau deposition drives neuropathological, inflammatory and behavioral abnormalities independently of neuronal loss in a novel mouse model
Source: Hum Mol Genet. 2015 Aug 13;24(21):6198–212. doi: 10.1093/hmg/ddv336 (PMC4599677; doi:10.1093/hmg/ddv336)
Supplement: Supplementary Data [file supp_24_21_6198__index.html]

Tau deposition drives neuropathological, inflammatory and behavioral abnormalities independently of neuronal loss in a novel mouse model — Tau deposition drives neuropathological, inflammatory and behavioral abnormalities independently of neuronal loss in a novel mouse model — Supplementary Data 

# Tau deposition drives neuropathological, inflammatory and behavioral abnormalities independently of neuronal loss in a novel mouse model

## Supplementary Data

Supplementary Data

- Supplementary Data - Pdf file
