# Supplementary material for: Tau deposition drives neuropathological, inflammatory and behavioral abnormalities independently of neuronal loss in a novel mouse model
Source: Hum Mol Genet. 2015 Aug 13;24(21):6198–212. doi: 10.1093/hmg/ddv336 (PMC4599677; doi:10.1093/hmg/ddv336)
Supplement: Supplementary Data [file supp_ddv336_ddv336supp.pdf]

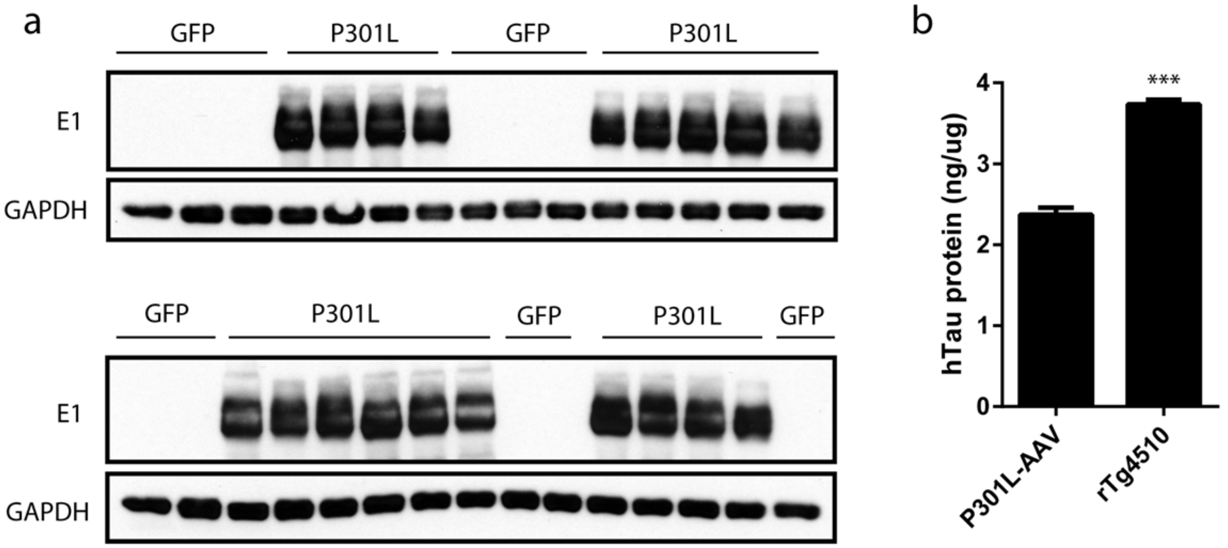

Figure S1. Low variability in human tau expression in Tau-AAV^{P301L} cohort. (**a**) Using an antibody specific for human tau (E1), the level of AAV1-Tau^{P301L} expression was measured in the SDS-soluble fraction from the forebrain of each animal. (b) Human tau protein levels were measured by MSD immunoassay (with E1 as the capture antibody, and Tau 5 as the detection) in AAV1-Tau^{P301L} and rTg4510 mice, and approximate concentrations (represented in ng of human tau per μg of brain tissue) were calculated using recombinant human tau to generate a standard curve. ***p<0.0001

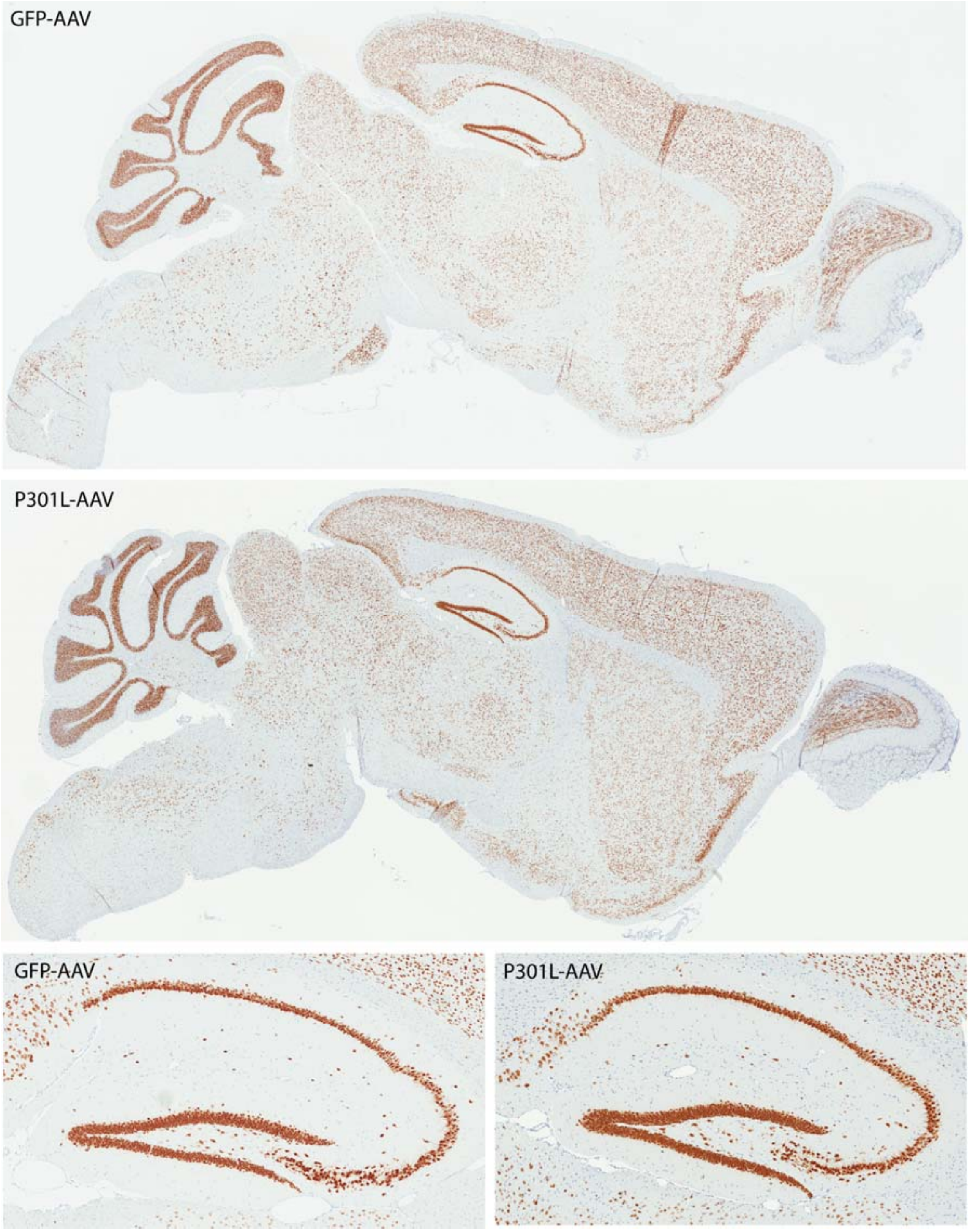

Figure S2. No change in cortical neuronal density in AAV1-Tau^{P301L} mice. To assess neuronal loss, the total number of NeuN-positive nuclei in the cortex was calculated and divided by the total area, as described (1). There was no significant difference in the number of NeuN-positive cells in the cortex between GFP-AAV and AAV1-Tau^{P301L} mice (t=1.47, p=0.17).

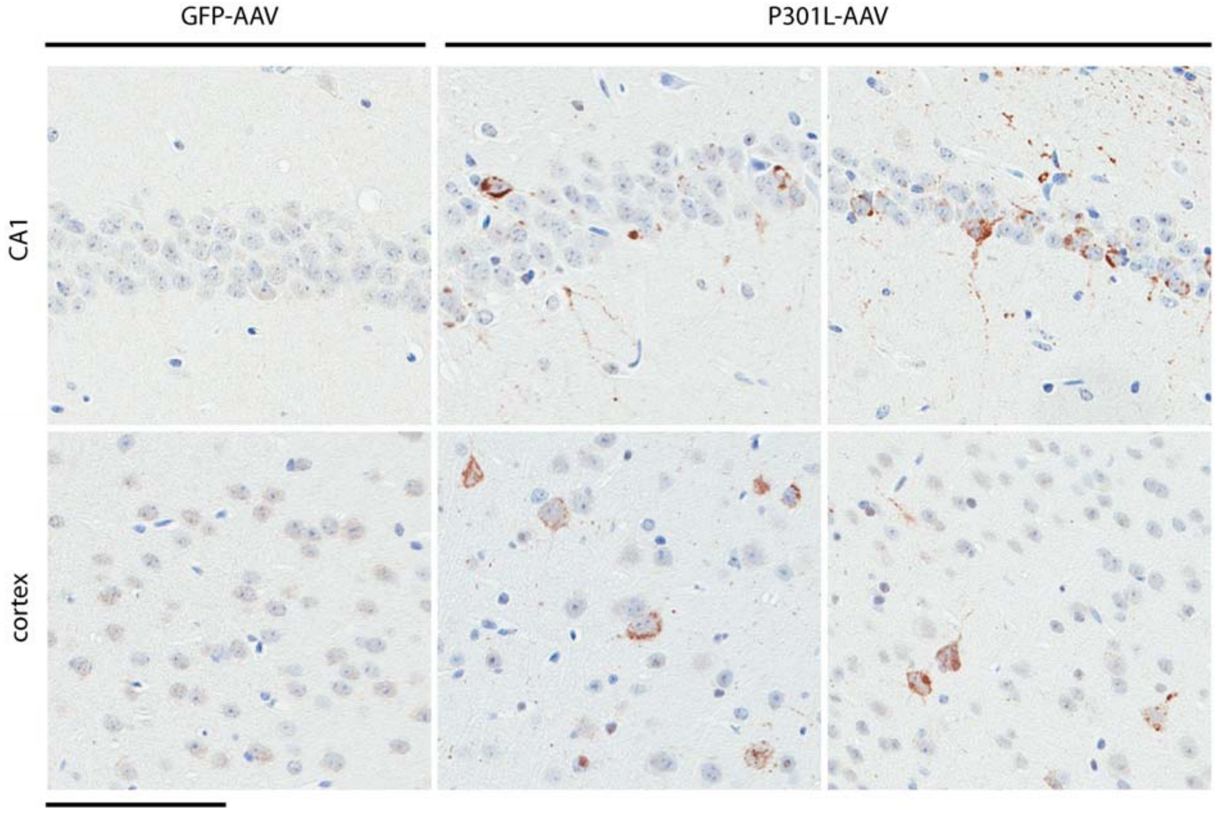

Figure S3. Tau inclusions are ubiquitinated. Overexpression of human P301L-tau drives the formation of inclusions that are positive for ubiquitin. Scale bar equal to 100μm.

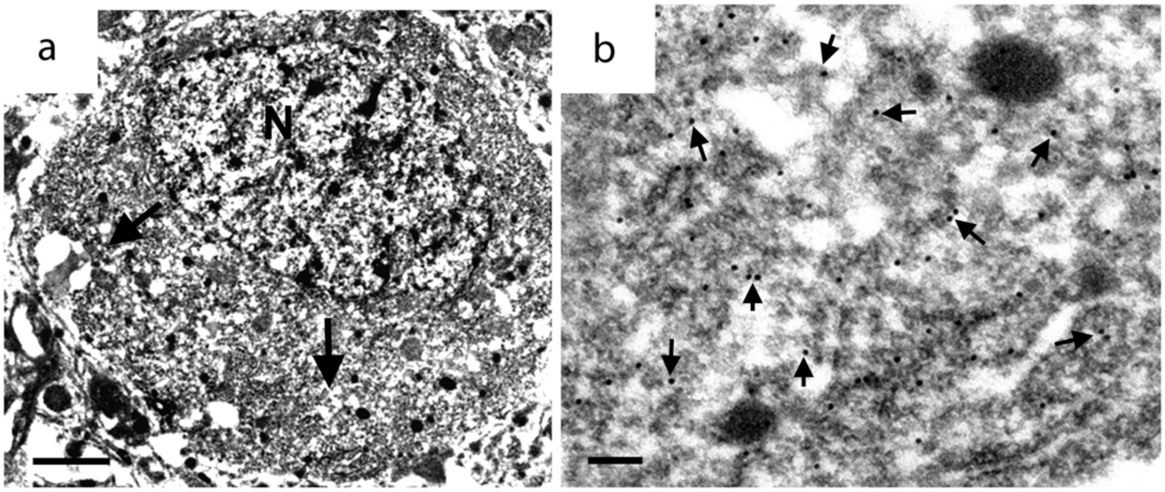

Figure S4. Immunogold labeling of tau pathology in hippocampal neurons. (**a**) A neuron has no filamentous aggregates, but has E1 labeling throughout the entire cell body. (**b**) Area enlarged in **(a)** revealed diffuse distribution of gold particles. Arrows point to 18 nm gold particles. N, nucleus. Scale bar equal to 0.5 µm (**a**); 100 nm (**b**).

References

1 Chew, J., Gendron, T.F., Prudencio, M., Sasaguri, H., Zhang, Y.J., Castanedes-Casey, M., Lee, C.W., Jansen-West, K., Kurti, A., Murray, M.E. *et al.* (2015) C9ORF72 repeat expansions in mice cause TDP-43 pathology, neuronal loss, and behavioral deficits. *Science*.
